# Epigenetic editing at individual age-associated CpGs affects the genome-wide epigenetic aging landscape

**DOI:** 10.1038/s43587-025-00841-1

**Published:** 2025-03-24

**Authors:** Sven Liesenfelder, Mohamed H. Elsafi Mabrouk, Jessica Iliescu, Monica Varona Baranda, Athanasia Mizi, Juan-Felipe Perez-Correa, Martina Wessiepe, Argyris Papantonis, Wolfgang Wagner

**Affiliations:** 1 Institute for Stem Cell Biology, RWTH Aachen University Medical School, Aachen, Germany; 2 Helmholtz-Institute for Biomedical Engineering, RWTH Aachen University Medical School, Aachen, Germany; 3 Interdisciplinary Centre for Clinical Research Aachen, RWTH Aachen University, Aachen, Germany; 4 Institute of Pathology, University Medical Center Göttingen, Göttingen, Germany; 5 Institute for Transfusion Medicine, Faculty of Medicine, RWTH Aachen University, Aachen, Germany; 6 Center for Integrated Oncology Aachen Bonn Cologne Düsseldorf (CIO ABCD), Aachen, Germany

**Keywords:** DNA methylation, DNA methylation, Predictive markers, Molecular engineering, Ageing

## Abstract

Aging is reflected by genome-wide DNA methylation changes, which form the basis of epigenetic clocks, but it is largely unclear how these epigenetic modifications are regulated and whether they directly affect the aging process. In this study, we performed epigenetic editing at age-associated CpG sites to explore the consequences of interfering with epigenetic clocks. CRISPR-guided editing targeted at individual age-related CpGs evoked genome-wide bystander effects, which were highly reproducible and enriched at other age-associated regions. 4C-sequencing at age-associated sites revealed increased interactions with bystander modifications and other age-related CpGs. Subsequently, we multiplexed epigenetic editing in human T cells and mesenchymal stromal cells at five genomic regions that become either hypermethylated or hypomethylated upon aging. While targeted methylation seemed more stable at age-hypermethylated sites, both approaches induced bystander modifications at CpGs with the highest correlations with chronological age. Notably, these effects were simultaneously observed at CpGs that gain and lose methylation with age. Our results demonstrate that epigenetic editing can extensively modulate the epigenetic aging network and interfere with epigenetic clocks.

## Abstract (web summary)

Using CRISPR-guided editing, Liesenfelder et al. modify DNA methylation at epigenetic clock sites, which evokes genome-wide age-associated epigenetic modifications. Their findings suggest age-associated DNA methylation changes can be coherently modified within an epigenetic network.

## Main

The epigenetic landscape changes continuously during human aging. This is particularly reflected by increased or decreased DNA methylation (DNAm) at specific CpG dinucleotides in the genome^1^. Due to the steady and reproducible nature of these changes epigenetic clocks are widely used as a biomarker for aging^2,3^. Accelerated epigenetic age is associated with higher all-cause mortality, indicating that these epigenetic signatures rather reflect biological than chronological age^4–7^. While age-associated DNAm changes are well characterized as a predictor, it is largely unclear how they are coherently modified across the genome and whether they directly affect the aging process^3,8,9^.

So far, the methods to reset epigenetic clocks are limited. Cells can be rejuvenated by reprogramming into induced pluripotent stem cells, but this involves a complete resetting of epigenetic and functional characteristics of the cells^10,11^. It is yet unclear whether partial reprogramming can stably interfere with epigenetic clocks, while maintaining cellular identity^12^. More recently, epigenetic editing approaches have been developed to facilitate site-specific modification of the DNAm pattern. For instance, the CRISPR toolbox uses single guide RNAs to direct a fusion protein of the nuclease-deficient Cas9 with the DNA methyltransferase DNMT3A/3L (dCas9-DNMT3A) to specific sites in the genome^13^. While DNMT3A-mediated epigenome editing is rather transient at most regions and often lost within a few days^14^, more complex fusion proteins have been developed for more sustained manipulations. For example, ‘CRISPRoff’ includes an additional Krüppel-associated box (KRAB) domain that increases the stability of DNA-hypermethylation^15–17^; however, it has been shown that dCas9-methyltransferases can have surprisingly ubiquitous nuclear activity with many potential off-target effects^18^.

So far, epigenetic editing has not been used systematically to interfere with epigenetic clocks, and this approach may seem counterintuitive, given that aging is reflected by genome-wide changes in the DNAm pattern. On the other hand, there may be crosstalk of epigenetic modifications within a network. For example, we have recently observed in acute myeloid leukemia that patient-specific aberrant DNAm patterns were always symmetric on both alleles, which may be governed by an inter-allelic epigenetic crosstalk^19^. Furthermore, age-associated DNAm seems to evolve coherently in single-cell analysis^20^. Some methylation changes seem to originate from CpG clusters that are coherently modified with age^21^. To further investigate the potential co-regulation of age-associated DNAm in a network-like manner, we conducted epigenetic editing at epigenetic clock sites. Our findings provide evidence that local changes in DNAm can result in a genome-wide response of aging signatures.

## Results

### CRISPR-guided epigenetic editing is site specific and stable

**Fig. 1:**
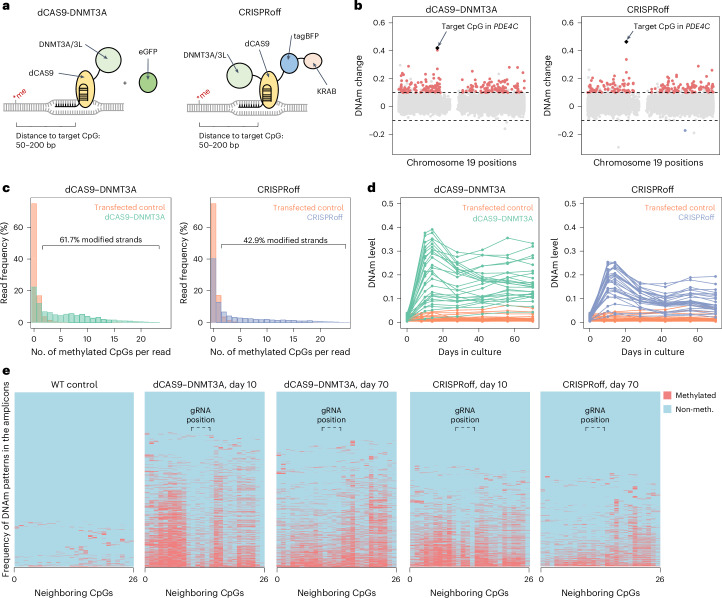
Epigenetic editing is stable but not coherent within the target region. **a**, Schematic presentation of two CRISPR-guided epigenetic editors used in this study. Deficient CAS9 protein is linked to DNMT3A/3L at the C terminus (dCAS9-DNMT3A/3L, coexpressed with eGFP for selection)^13^, and CRISPRoff comprising DNMT3A/3L at the N terminus, and tagBFP and a KRAB domain at the C terminus^15^. Single guide RNAs were designed 50–200 bp distant of the target CpG. **b**, Manhattan plot illustrating the DNAm changes upon targeting the age-associated hypermethylated region in *PDE4C* (EPIC Illumina BeadChip data of chromosome 19; mean of three replicas; 14 days after transfection). Significant DNAm changes are highlighted in red: delta mean DNAm > 0.1 and an adj. *P* < 0.05 (limma *P* value, Benjamini–Hochberg adjusted). The target CpG in *PDE4C* is highlighted in black. **c**, Bisulfite amplicon sequencing of 26 neighboring CpGs at the target region of *PDE4C*. The bar plot depicts the frequency of methylated CpGs on individual reads, indicating that even on modified DNA strands not all neighboring CpGs become coherently methylated. 61.7% and 42.9% of reads have higher methylation levels than observed in the wild type, for dCAS9-DNMT3A and CRISPRoff, respectively. **d**, Time-course experiment of DNAm at *PDE4C* measured by bisulfite barcoded amplicon sequencing. The lines resemble the 26 different CpGs in the amplicons. **e**, Frequency of DNAm patterns in bisulfite barcoded amplicon sequencing data of *PDE4C*. Reads are clustered by their DNAm pattern. The binding region of one gRNA is indicated. The second gRNA binds two base pairs next to the CpG #26 and might explain the low methylation gain at #25 and #26.

**Extended Data Fig. 1:**
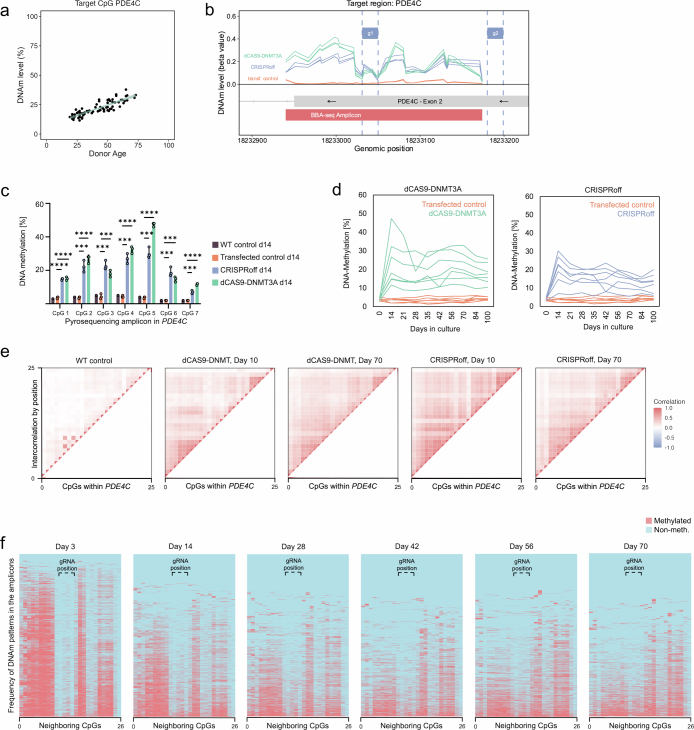
DNA methylation in amplicons of the target region. **a**) DNA methylation at the most age-associated CpG site in *PDE4C* was measured by digital droplet PCR in peripheral blood, as described before (n = 80, R^2^ = 0.76; Han et al., *BMC Biology* 18,71 (2020)). **b**) Schematic presentation of the genomic region in *PDE4C* with guide RNA binding sites (blue) and the bisulfite amplicon sequencing region with corresponding methylation changes. **c**) Pyrosequencing measurement of HEK293T cells after epigenetic modification at *PDE4C* (three replicates). DNAm levels at the seven CpGs in the pyrosequencing amplicon are depicted at day 14 (***P < 0.001; **** P < 0.00001). **d**) Pyrosequencing of the same samples over a time course of up to 100 days confirmed that the modified DNAm is stable at this site. **e**) Correlation of DNAm was analyzed at the 26 neighboring CpGs within the bisulfite barcoded amplicon sequencing reads. The heatmap depicts Pearson correlations between individual CpGs. f) Heatmaps showing the complete time-course experiment upon modification with dCAS9-DNMT3A, corresponding to Fig. 1e.

Initially, we aimed to modify DNAm in a genomic region within phosphodiesterase 4C (*PDE4C)*, which was identified as one of the first sites to gain age-associated DNAm across different tissues^1^ and has a very high correlation with chronological age in blood^11^ (Extended Data Fig. 1a). For initial experiments, we used HEK293T cells, as they resemble a standardized, homogenous and relatively easy to handle cell line. While the epigenetic age of HEK293T cells does not reflect the chronological age of the donor, due to clonal derivation, immortalization and in vitro expansion, the cell line may still provide insight into coherent age-associated DNAm changes^22^. We used two alternative constructs to modulate the target region in *PDE4C*: either dCAS9-DNMT3A^13^ or CRISPRoff^15^ (both *n* = 3), each with two guide RNAs for the *PDE4C* locus (Fig. 1a and Extended Data Fig. 1b). Pyrosequencing validated a highly significant increase in DNAm across all seven CpGs in this amplicon (*P* < 0.001; *n* = 3; Extended Data Fig. 1c). Furthermore, Illumina BeadChip (EPIC) analysis 14 days after transfection showed the highest gain of DNAm at the target site in *PDE4C* (Fig. 1b). Both methods revealed up to 40% gain of DNAm at individual CpGs. Thus, the mean changes in DNAm are in a similar range as age-associated DNAm changes at the *PDE4C* locus. Either way, as each individual cell has only two copies of DNA, the DNAm levels at a single-cell level can only be 0%, 50% and 100%, both upon epigenetic editing and aging.

To get better insight into the DNAm changes at this region, we performed bisulfite amplicon sequencing that covered 26 neighboring CpGs. Anticipating that the fraction of modified DNA strands might be even higher than site-specific DNAm changes, we analyzed the frequency of methylated CpGs in individual reads: we hardly observed two or more methylated CpGs in the controls, whereas 61.7% and 42.9% of reads showed higher methylation levels in dCAS9-DNMT3A and CRISPRoff modified cells, respectively (Fig. 1c). This demonstrates that due to heterogeneity of DNAm at neighboring CpGs epigenetic editing was even more efficient than indicated by crude DNAm levels at individual sites. Furthermore, hypermethylation was stable over at least 100 days for both dCAS-DNMT3A and CRISPRoff, even though the cells were highly proliferative and lost the transiently transfected plasmids within few days (Fig. 1d and Extended Data Fig. 1d).

Initially we expected that when a CRISPR-DNMT3A construct is targeted to a specific genomic region, all neighboring CpGs should be coherently modified; however, as mentioned above, the bisulfite barcoded amplicon sequencing data demonstrated that modifications at the 26 neighboring CpGs in the amplicon were overall not coherently modified (Extended Data Fig. 1e). Particularly with the dCAS9-DNMT3A construct, we observed less gain of DNAm at guide RNA binding positions (Fig. 1e). Notably, during the time course, this lowly methylated region assimilated toward the higher DNAm at neighboring CpGs with time (Extended Data Fig. 1f). This indicates local DNAm levels within the target region become more homogeneous over several months, albeit the transient CRISPR-guided DNA methyltransferases are no longer present.

### Epigenetic editing evokes genome-wide bystanders

**Fig. 2:**
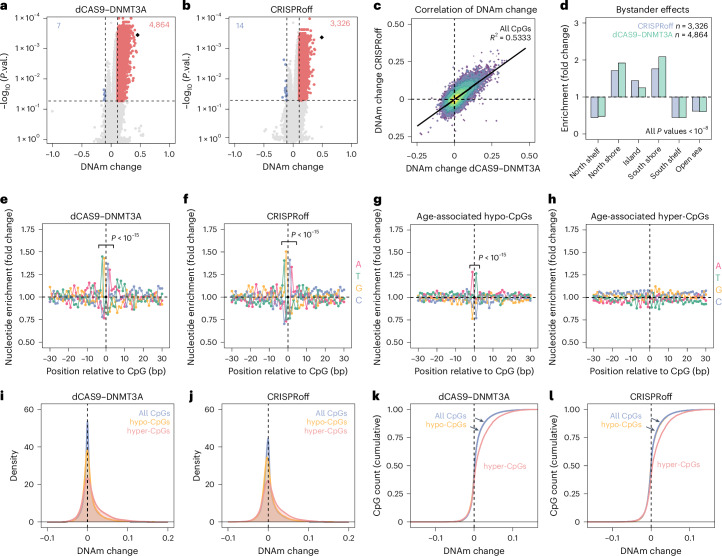
Genome-wide bystander effects of epigenome editing. **a**,**b**, Significant DNAm changes upon epigenetic editing at *PDE4C* with either dCas9-DNMT3A (**a**) or CRISPRoff (**b**). Volcano plots depict significantly hypermethylated (red) and hypomethylated (blue) CpGs and their numbers are indicated (*n* = 3; 14 days after transfection; limma *P* value, Benjamini–Hochberg adjusted). **c**, Multivariate density estimate comparing DNAm changes in the dCAS9-DNMT3A and CRISPRoff experiments. Pearson correlation (*R*^2^ = 0.53) indicates that there is a high reproducibility of the bystander modifications, even with the different epigenetic modification approaches. **d**, Enrichment of the bystander effects in relation to CpG islands (CGI), and at shore and shelf regions surrounding CGIs. Enrichment was calculated in relation to all CpGs on the array and was highly significant for all categories: chi-squared test, degrees of freedom (d.f.) = 1, not adjusted for multiple testing, North Shelf *P* = 9.51 × 10^−10^ (CRISPRoff) and *P* = 2.80 × 10^−12^ (DNMT3A), South Shelf *P* = 4.76 × 10^−9^ (CRISPRoff) and *P* = 8.79 × 10^−13^ (DNMT3A), all other *P* < 10^−15^. **e**,**f**, Relative frequency of nucleotides next to CpGs with epigenetic bystander modifications normalized to CpGs with identical CpG density (EPIC manifest). Guanine and cytosine were overrepresented at the −1 and +1 flank positions, whereas thymine and adenine were enriched at the −2 and +2 positions (separate chi-squared tests of the four proximate genomic positions, d.f. = 1, not adjusted for multiple testing, all four *P* < 10^−15^). **g**,**h**, Relative frequency of nucleotides next to CpGs that become either hypomethylated with age (**g**; 4,389 CpGs), or hypermethylated with age (**h**; 5,328 CpGs) in a large-scale epigenome-wide association study^25^. Frequency was normalized to the entirety of CpGs on the BeadChip. Adenine and thymidine were overrepresented in the −1 and +1 flank positions of hypomethylated CpGs (separate chi-squared tests of the two proximate genomic positions, d.f. = 1, not adjusted for multiple testing, both *P* < 10^−15^). **i**,**j**, Distribution of epigenetic bystander modifications in the dCAS9-DNMT3A experiments (**i**) and the CRISPRoff experiments (**j**) was analyzed for all CpGs on the array, for the 4,389 CpGs with age-associated hypomethylation and 5,328 CpGs with age-associated hypermethylation^25^. Age-associated hypermethylation was enriched at CpGs that gain DNAm upon epigenetic editing (both *P* < 10^−15^, two-sided, two-sample Kolmogorov–Smirnov test). **k**,**l**, Cumulative distribution of the density functions in **i**,**j** to better visualize that bystander effects upon targeting *PDE4C* are enriched at other age-hypermethylated CpGs.

**Extended Data Fig. 2:**
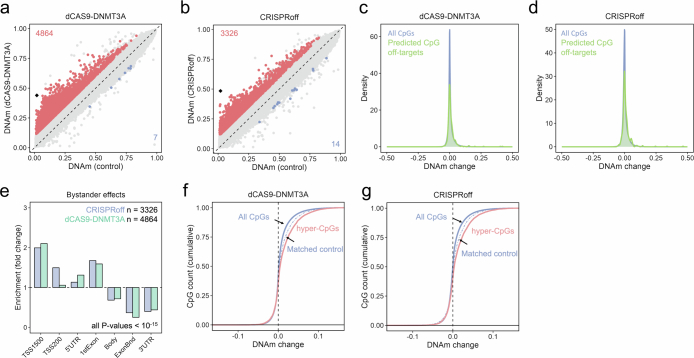
Further analysis of epigenetic bystander modifications. **a,b**) Scatterplots of Illumina BeadChip data demonstrate DNAm changes in HEK293T cells upon targeted methylation at *PDE4C* with either dCas9-DNMT3A (a; n = 3), or CRISPRoff (b; n = 3). Significant CpGs are highlighted in analogy to Fig. 2a, b. **c,d**) Due to sequence homology, 123 genomic regions were identified as potential off-target binding sites. Within a window of 5 kb, we identified 300 CpGs in these regions. Predicted off-target binding sites for *PDE4C* gRNAs did not reveal clear enrichment in bystander modifications of dCas9-DNMT3A (c), or CRISPRoff (d). **e**) Enrichment of the bystander effects in relation genomic regions: TSS1500: 1500 bp upstream of transcription start site (TSS); TSS200: 200 bp upstream of TSS; UTR: untranslated region. Enrichment was calculated in relation to all CpGs on the array and was highly significant for all categories (Chi^2^ test). **f,g**) Cumulative distribution function analog to Fig. 2k, l comparing DNAm profiles of all CpGs, age-hypermethylated CpGs and a matched control, which has the same distribution of beta-values as age-hypermethylated sites in HEK293T control cells. Enrichment is also highly significant accounting for beta-values (Fig. f: P = 7.57*10^−10^; Fig. g: P = 2.59*10^−10^, two-sided, two-sample Kolmogorov–Smirnov-test).

While the highest gains of DNAm were observed at the target site, there were also many off-target effects. When we analyzed EPIC BeadChip data of the three independent replicas, we observed significant increases of DNAm (abs. diff. *β* > 0.1 and *P* value < 0.05) at 4,864 sites and 3,326 sites for dCAS9-DNMT3A and CRISPRoff, respectively (Fig. 2a,b and Extended Data Fig. 2a,b). Furthermore, there was even a significant decrease in DNAm at either 7 or 14 CpGs upon epigenome editing with these methods, which is in line with previous observations^23^ and may not be explained by the activity of the constructs. Direct comparison of DNAm changes with dCAS9-DNMT3A and CRISPRoff revealed a correlation of *R*^2^ = 0.53 for all CpGs, indicating that the gains of methylation are not just random footprints of the constructs, but rather consistent epigenetic bystander modifications (Fig. 2c). Notably, even the hypomethylation was rather consistent with both editors.

Subsequently, we tested whether the bystander effects were enriched at genomic regions that were predicted to have potential CRISPR-mediated off-target effects. Based on sequence homologies to our guide RNAs 123 regions were predicted to be potential off-target sites, but we did not observe any methylation changes here (Extended Data Fig. 2c,d). Instead, hypermethylated bystanders were ubiquitously enriched in islands and shores, but decreased in the shelf and open sea (*P* < 10^−9^; Fig. 2d). Bystander effects were rather observed in upstream promotor regions (Extended Data Fig. 2e). Furthermore, the frequency of nucleotides at neighboring positions of bystander effects featured distinct enrichment: Guanine and cytosine were present at the −1 and +1 positions about 37–50% more often than for all CpGs. Besides, thymine and adenine were enriched at the −2 and +2 positions, respectively (Fig. 2e,f). This nucleotide pattern around bystander CpGs has a moderate association with the flank sequence preferences of DNMT3A^24^.

### Bystander modifications are enriched at other age-associated CpGs

Next, we sought to explore whether the epigenetic bystander modifications upon targeting *PDE4C* are potentially related to other epigenetic clock sites. Albeit we used HEK293 cells in these experiments, we exemplarily focused age-associated DNAm changes in blood samples as age-associated DNAm is often observed at similar genomic regions across different cell types^1,10^. We utilized results of a large-scale epigenome-wide association study of 18,413 individuals that identified the top 10,000 CpGs linearly associated with age^25^. Of these, 5,328 hypermethylated and 4,389 hypomethylated CpGs passed the filter criteria for our analysis. Flank sequences of age-associated sites did not reveal a clear motive for age-associated hypermethylation, whereas the hypomethylated CpGs showed a complementary enrichment of adenine and thymine at the −1 and +1 position, which was in line with previous reports^26^ (Fig. 2g,h).

Subsequently, we tested whether the bystander DNAm changes upon modification of *PDE4C* were enriched in age-associated hyper- or hypomethylated CpGs. Of note, the 5,328 CpGs with age-associated hypermethylation showed a significant methylation gain compared to all other CpGs, with either dCAS9-DNMT3A or CRISPRoff (*P* < 10^−15^, Fig. 2i–l). This effect was still significant, when accounting for lower *β*-values of age-hypermethylated CpGs in HEK293T cells (Extended Data Fig. 2f,g), indicating that the epigenetic bystander modifications of *PDE4C* are associated with other age-associated DNAm changes.

**Extended Data Fig. 3:**
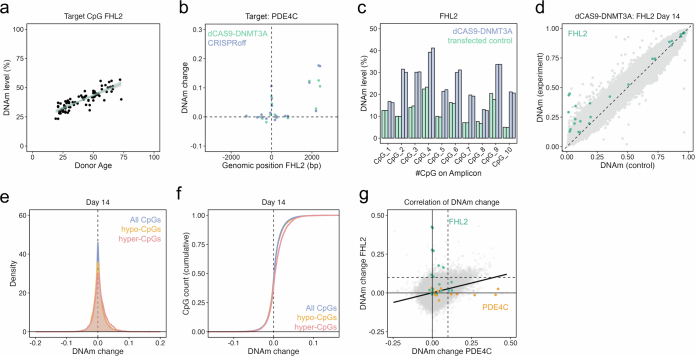
Epigenetic editing at *FHL2.* **a**) Methylation at the CpG site cg22454769 in *FHL2* reveals a strong correlation with chronological age in blood, as previously described (n = 80, R^2^ = 0.72; Han et al., *BMC Biology* 18,71 (2020)). **b**) The CpG site cg22454769 in *FHL2* (centered at genomic position 0) revealed gain of DNAm upon targeting *PDE4C* with either CRISPRoff or dCAS9-DNMT3A (Illumina BeadChip data; both n = 3). **c**) The genomic region in *FHL2* was targeted with dCAS9-DNMT3A in HEK293T cells and successful gain in DNAm was demonstrated by pyrosequencing after 14 days (cg22454769 corresponds to CpG 2; n = 2). **d**) Scatter-plot of EPIC BeadChip data confirming the specificity of epigenetic editing at *FHL2* (CpGs within a 10 kbp window around cg22454769 are highlighted; n = 2). **e,f**) Gaussian kernel density estimate and cumulative distribution function showing enrichment of bystander effects at 5076 age-hypermethylated and 4127 age-hypomethylated sites (P < 10^−15^ for age-hypermethylated and P = 0.0009 age-hypomethylated CpGs; two-sided, two-sample Kolmogorov–Smirnov-test). **g**) Scatter-plot of EPIC datasets comparing methylation changes as compared to corresponding controls after targeting either *PDE4C* or *FHL2* with dCAS9-DNMT3A (R = 0.32; P < 10^−15^, Pearson correlation test). The dotted lines indicate at least 10% gain of DNAm upon targeting either *PDE4C* or *FHL2*, and 488 CpGs passed both thresholds.

To further validate that targeting of age-associated CpGs affects DNAm at other age-associated regions, we have alternatively targeted a region in the gene Four and a half LIM domains protein 2 (*FHL2*), which is also highly age-correlated^27^ and showed bystander effects in the previous *PDE4C* experiment (Extended Data Fig. 3a,b). Successful modification of DNAm in *FHL2* with dCAS9-DNMT3A in HEK293T cells was again demonstrated by pyrosequencing and EPIC BeadChip analysis (Extended Data Fig. 3c,d). At 14 days after transfection, there was a significant gain of DNAm at age-hypermethylated sites (*P* < 10^−15^; Extended Data Fig. 3e,f). Furthermore, there was a moderate correlation of genome-wide methylation effects when targeting either *FHL2* or *PDE4C* (Extended Data Fig. 3g).

### Chromatin interactions contribute to bystander effects

**Fig. 3:**
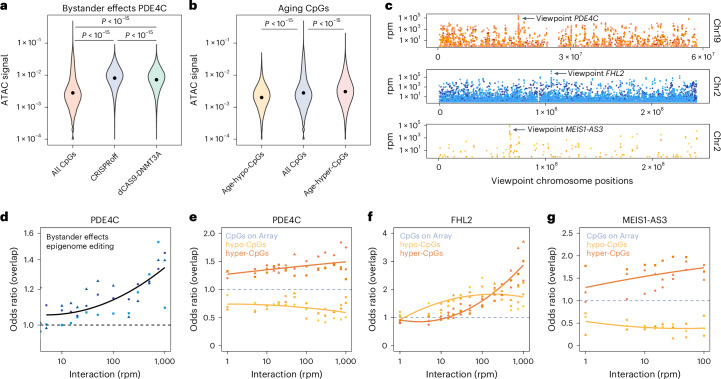
Chromatin conformation contributes to epigenetic bystander modifications. **a**, ATAC-seq data of HEK293T cells^28^ was used to estimate chromatin accessibility at epigenetic bystander modifications upon modification of *PDE4C* with either dCas9-DNMT3A or CRISPRoff. The higher ATAC-seq signals indicate that bystander modifications are enriched at open chromatin regions (two-tailed, unpaired *t*-test). **b**, In analogy, we tested whether CpGs with age-associated DNAm changes are also associated to chromatin accessibility in the ATAC-seq data HEK293T cells. The 4,389 age-hypomethylated CpGs revealed overall lower ATAC signal, whereas the 5,328 age-hypermethylated CpGs were enriched at open chromatin (two-tailed unpaired *t*-test). **c**, Intrinsic 4C-sequencing in HEK293T cells for three age-associated sites in *PDE4C, FHL2* and *MEIS1-AS3* (triplicates per viewpoint). The Manhattan plots depict the number of reads (rpm, reads per million) at the *cis*-interacting regions on the corresponding chromosomes. The highest signal was observed around the 4C viewpoints. **d**, Bystander effects of *PDE4C* become enriched with increasing coverage in 4C-sequencing of *PDE4C-*interacting sites. The association is best described by an exponential model (adjusted *R*^2^: 0.586, model *P* value < 1.801 × 10^−7^). Different symbols correspond to the three replicates. **e**–**g**, Overlap of 4,389 age-hypo and 5,328 age-hypermethylated CpGs at interacting genomic regions in 4C-sequencing data (chi-squared tests were performed for the threshold of 100 rpm: *PDE4C*: age-hypermethylated sites *P* = 0.0004 and age-hypomethylated sites *P* = 0.0019 (**e**); *FHL2*: age-hypermethylated sites *P* = 0.0008 and age-hypomethylated sites *P* = 0.0088 (**f**); and *MEIS1-AS3*: age-hypermethylated sites *P* = 0.0008 and age-hypomethylated sites *P* = 0.009 (**g**); all d.f = 1).

**Extended Data Fig. 4:**
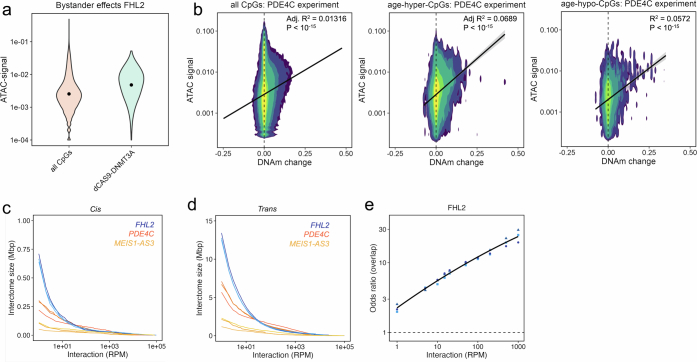
Further analysis of ATAC-seq and 4C-sequencing data. **a**) Violin-plot of ATAC-seq signals comparing chromatin accessibility at all CpGs and hypermethylated bystander positions of *FHL2* (delta DNAm >10%; n = 2, P < 10^−15^, unpaired t-test), in analogy to Fig. 3a. **b**) Multivariate kernel density estimate of ATAC-seq and DNA methylation changes (dCAS9-DNMT3A). While differentially methylated positions exhibited in tendency higher chromatin accessibility the overall correlation was rather low. In analogy, we analyzed the 4389 age-hypo and 5328 age-hypermethylated CpGs. **c**) Cis-interactions (on the same chromosome) of 4C-Sequencing with around 20 million reads per sample. Particularly the two viewpoints at positively age-correlated regions in *PDE4C* and *FHL2* exhibited cis-interactions spanning a mean of 271 and 672 kbp, respectively. In contrast, the 4 C reads of age-hypomethylated region in *MEIS1-AS3* comprised only 89 kbp. **d**) Trans-interactions (across different chromosomes) of 4C-sequencing. The viewpoints at positively age-correlated regions like *PDE4C* and *FHL2* exhibited trans-interactions spanning a mean of 6.5 and 13.0 mbp, respectively. **e**) Bystander effects of *FHL2* (abs. difference beta-value > 0.1; n = 2) become enriched with increasing coverage in 4C-sequencing of *FHL2* interacting sites (n = 3, different symbols correspond to replica; R^2^ = 0.81, linear model P < 10^−15^).

To better understand why the bystander modifications occur in this highly reproducible manner, we investigated transposase-accessible chromatin with high-throughput sequencing (ATAC-seq) data of HEK293T cells^28^. In fact, the epigenetic bystander modifications upon targeting *PDE4C* or *FHL2* were clearly enriched at open chromatin (Fig. 3a and Extended Data Fig. 4a). In contrast, age-hypomethylated CpGs were rather associated with closed chromatin (Fig. 3b). Conversely, age-hypermethylated CpGs tended to have higher chromatin accessibility, indicating that some bystanders might be attributed to chromatin accessibility; however, performing logistic regression analysis on methylation changes and chromatin accessibility this association was less evident, indicating that chromatin state alone does not sufficiently explain the bystander effects with epigenome editing (Extended Data Fig. 4b; *R*^2^ = 0.0689 for hyper-CpGs and *R*^2^ = 0.0572 for hypo-CpGs).

Next, we investigated whether the coherent modification of bystander DNAm changes might also be attributed to the higher order of chromatin. Therefore, we conducted intrinsic 4C-sequencing to identify chromatin that interacted with the target sites in *PDE4C* or *FHL2*. For comparison, we considered a hypomethylated region in MEIS1 antisense RNA 3 (*MEIS1-AS3;* all *n* = 3; Extended Data Fig. 4c,d). As expected, signals with the highest read-per-million (rpm) counts were localized close to the viewpoints (Fig. 3c). Excluding the *PDE4C* target region from analyses, we observed that bystander modifications (abs. diff. *β* > 0.1 and *P* < 0.05) were increasing with higher interaction to *PDE4C* (Fig. 3d). This association was best described by exponential modeling for the highly interacting CpGs. Accordingly, bystander effects of *FHL2* were increasing with interaction strength to the *FHL2* viewpoint (Extended Data Fig. 4e). Therefore, at least some of the bystander modifications could be attributed to interacting chromatin.

To explore whether age-associated genomic regions might be generally enriched at interacting chromatin, we analyzed the overlap of the age-hyper- and hypomethylated CpGs with 4C reads. In fact, particularly the CpGs that become hypermethylated with aging were enriched in the interactome of all three age-associated CpGs (Fig. 3e–g). Considering that *PDE4C* and *FHL2* revealed larger interactomes as compared to *MEIS1-AS3* (Extended Data Fig. 4c,d), it seems that particularly CpGs with age-associated hypermethylation seem to have chromatin interaction with each other. Overall, the spatial interaction of epigenetic bystander modifications and age-associated CpGs further pointed toward an interacting epigenetic network.

### Multiplexed epigenetic editing at age-associated CpGs

**Fig. 4:**
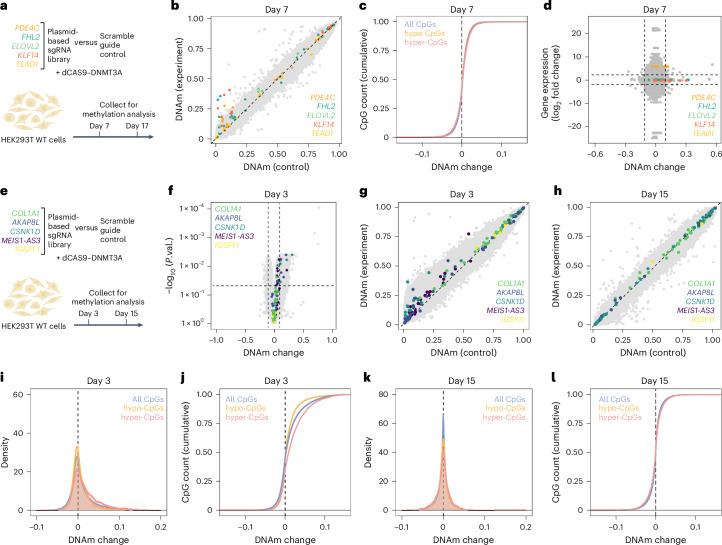
Multiplexed epigenetic editing at age-hyper- and age-hypomethylated genomic regions. **a**, Scheme of multiplexed epigenetic editing at five genomic regions that gain DNAm with aging. **b**, Scatter-plot of DNAm changes across the three replicas. CpGs corresponding to the targeted genes are highlighted in the corresponding color. None of the bystander modifications reached statistical significance. **c**, Cumulative distribution function comparing the entirety of CpGs from the BeadChip with age-associated CpGs (4,389 age-hypo and 5,328 age-hypermethylated CpGs). **d**, Correlation of differential gene expression with differential methylation. CpGs and transcripts were matched by gene IDs. Pairs related to one of the five target genes are highlighted in the corresponding color. **e**, Scheme of multiplexed epigenetic editing at five genomic regions that lose DNAm with aging. **f**,**g**, Three days after transfection, DNAm changes were analyzed by a volcano plot (**f**) and a scatter-plot (**g**). The volcano plot shows DNAm changes and the limma *P* value (Benjamini–Hochberg adjusted). CpGs corresponding to the five target genes are highlighted by the corresponding colors (*n* = 3). **h**, At 15 days after transfection, the gains in DNAm in the target regions were hardly observed anymore. **i**, At day 3 after transfection, Gaussian kernel density estimate at age-associated CpGs (4,389 age-hypo and 5,328 age-hypermethylated CpGs) showed that bystander modifications were enriched at genomic regions that gain methylation with age. **j**, Cumulative distribution of DNAm at age-associated CpGs at day 3 after transfection. Notably, bystander effects are underrepresented at age-hypo and overrepresented at age-hypermethylated CpGs (both *P* < 10^−15^, two-sided, two-sample Kolmogorov–Smirnov test). **k**,**l**, At day 15 after transfection kernel density estimate and cumulative distribution did not reveal bystander effects anymore.

**Extended Data Fig. 5:**
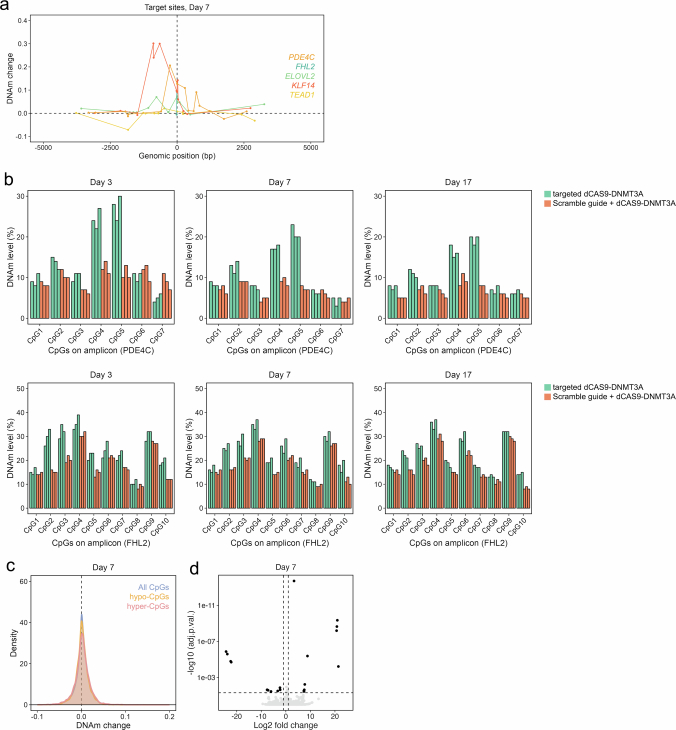
Pyrosequencing validation of targeted epigenetic modification. **a**) Genomic tracks of differential DNAm at day 7 in a 10k base window centered at the corresponding target site (dashed vertical line) in the five targeted genes. **b**) Pyrosequencing measurements of demonstrating stable DNAm levels at *PDE4C* and *FHL2* (triplicates). HEK293T cells were either transfected guides for *PDE4C, FHL2, ELOVL2, KLF14*, and *TEAD1* together with dCas9-DNMT3A, or scramble guide RNA and dCas9-DNMT3A. The epigenetic modifications both regions were stable for at least 17 days. DNAm was significantly higher in experimental conditions across all timepoints (*P* value < 0.05, unpaired t-test). **c**) Gaussian kernel density estimate comparing the entirety of CpGs from the BeadChip with age-associated CpGs (4389 age-hypo and 5328 age-hypermethylated CpGs). In these experiments hardly any reproducible bystander effects were observed. **d**) Transcriptomic changes upon multiplexed epigenetic editing at the age-hypermethylated CpGs. 10 genes were significantly up- and 11 genes significantly down-regulated (adjusted *P* < 0.05; >2 fold change).

We reasoned that targeting multiple age-associated CpGs might increase interferences with a possible epigenetic aging network. Therefore, we multiplexed targeting of five age-hypermethylated regions: *PDE4C, FHL2, ELOVL2, KLF14* and *TEAD1*. These CpGs were selected because of their high correlation of DNAm with chronological age^27,29^, low methylation in HEK293T cells and blood cells and ease to design specific guide RNAs. Initially, the experiments were again performed in HEK293T cells with a scramble guide RNA for control (*n* = 3; Fig. 4a). Seven days after transfection, EPIC Illumina BeadChip analysis demonstrated clear gains of DNAm at regions in *PDE4C, FHL2* and *KLF14* (Fig. 4b). These changes were distributed in a 1.5-kb area and remained stable for at least 17 days (Extended Data Fig. 5a,b); however, the site-specific gain of DNAm was less pronounced than previously observed and none of the bystander modifications reached statistical significance. Furthermore, we did not observe enrichment at age-associated regions in these experiments (Fig. 4c and Extended Data Fig. 5c). Transcriptomic analysis of these samples by RNA-sequencing revealed moderate but significant up- and downregulation of 10 and 11 genes, respectively (*n* = 3; adjusted *P* < 0.05; Extended Data Fig. 5d). Overall, there was no clear correlation between gene expression and DNAm changes. However, the target gene *PDE4C*, which is hardly expressed in HEK293T cells, revealed 46-fold higher expression upon multiplexed epigenome editing (not significant; Fig. 4d).

### Resetting DNA methylation at age-hypomethylated CpGs

**Extended Data Fig. 6:**
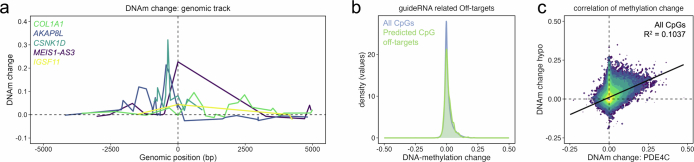
Targeted modifications at age-hypomethylated regions. **a**) Genomic tracks of differential DNAm at day 3 in a 10k base window centered at the corresponding target site (dashed vertical line) at five CpGs that lose DNAm with age. **b**) Kernel density estimate revealing no methylation changes at 692 predicted off-target binding sites (sequence homology) compared to all CpGs on BeadChip. **c**) Multivariate kernel density estimate and linear regression (Pearson correlation) comparing DNAm changes with epigenome editing between *PDE4C* and hypo-CpGs.

We next attempted to restore DNAm at five genomic sites that lose DNAm with age. To this end, we multiplexed guide RNAs for *COL1A1, AKAP8L, CSNK1D, MEIS1-AS3* and *IGSF11* (refs. ^27,29^), as compared to scramble guide RNAs for control (*n* = 3; Fig. 4e). Initial experiments indicated that modulation of CpGs that become hypomethylated with aging might be less stable and therefore we analyzed DNAm profiles already after 3 days. Many CpGs gained methylation including the target regions of *AKAP8L*, *CSNK1D* and *MEIS1-AS3* (Fig. 4f,g and Extended Data Fig. 6a); however, after 15 days these targeted gains of DNAm were already lost (*n* = 3; Fig. 4h). Thus, DNAm gains at age-hypomethylated regions seem to be less stable than at age-hypermethylated CpGs.

By day 3, there were 19,254 significant hypermethylated and 88 hypomethylated bystander effects (abs. diff. *β* > 0.1 and *P* value < 0.05). Bystanders were again not enriched in predicted guide RNA-related off-target regions (Extended Data Fig. 6b). Correlating DNAm changes to the initial *PDE4C* experiment, some of the genome-wide differences were consistent, but several changes were exclusively observed when targeting the five age-hypomethylated regions. This substantiates the notion that bystander modifications are not only footprints that are attributed to binding preferences of DNA methyltransferase domains (Extended Data Fig. 6c).

Subsequently, we tested whether the day 3 bystander modifications of the five age-hypomethylated target regions interfere with other age-associated sites. Notably, age-hypermethylated CpGs were again enriched in these bystanders, whereas the overlap with age-hypomethylated CpGs was significantly lower than expected (Fig. 4i,j). This was somewhat surprising as we have now modified CpGs that become hypomethylated with aging, but it clearly demonstrated that age-associated DNAm is coherently mediated, particularly at age-hypermethylated CpGs; however, by day 15 there were no longer any significant bystander modifications and, thus, also age-associated CpGs did not reveal consistent modifications (Fig. 4k,l).

### Epigenetic editing affects the aging network in T cells

**Fig. 5:**
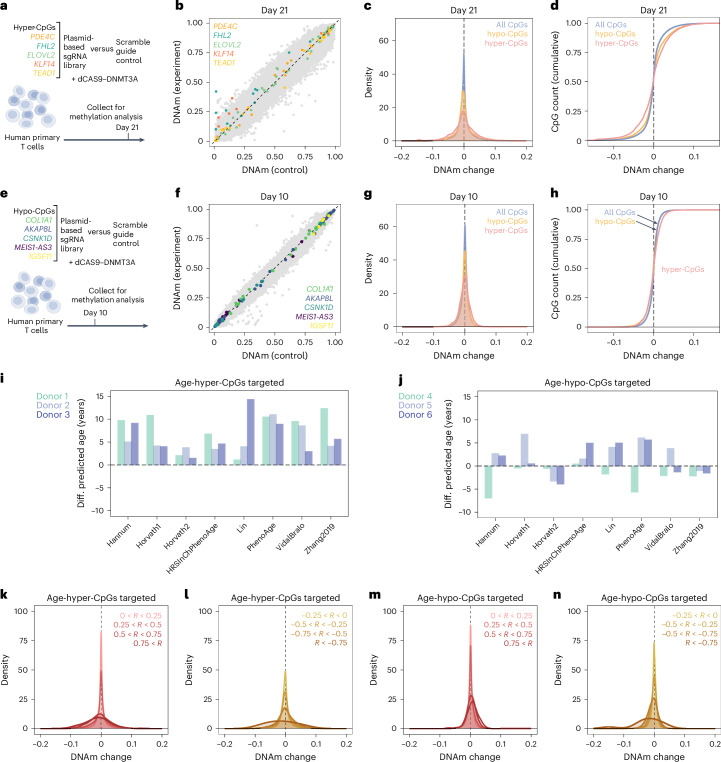
CRISPR-epigenetic editing in human primary T cells. **a**, Scheme of multiplexed epigenetic editing at five genomic regions that gain DNAm with aging. **b**, Scatter-plot of Illumina BeadChip data showing clear mean DNAm changes at *FHL2* and *KLF14* (*n* = 2). **c**,**d**, Gaussian kernel density estimates and cumulative distribution function showing significantly different methylation profiles at hyper- and hypo-CpGs (two-sided, two-sample Kolmogorov–Smirnov test *P* < 10^−15^). **e**, Scheme of multiplexed epigenetic editing at five genomic regions that lose DNAm with aging. **f**, Scatter-plot of Illumina BeadChip data revealing no mean methylation change after 10 days (*n* = 3). **g**,**h**, Gaussian kernel density estimates and cumulative distribution function. The genome-wide epigenetic landscape is less affected when targeting age-hypomethylated CpGs (10 days of culture). **i**,**j**, Estimation of epigenetic age with eight different epigenetic clocks:: Zhang^33^, Vidal-Bralo^34^, Lin^4^, Horvath 1 (multi-tissue)^10^, Horvath 2 (skin and blood)^35^, Hannum^29^ and PhenoAge and updated PhenoAge^7^. Bar plots depict the deviation of epigenetic age predictions upon targeted modification at the age-hypermethylated (**i**) and the age-hypomethylated CpGs (**j**), as compared to the scramble guide RNA controls (difference in years). **k**–**n**, Density plots of DNAm profiles for different strata of age-relatedness. Pearson correlation of individual CpGs with chronological age was determined in four T cell datasets^36–39^. Upon targeting the hyper-CpGs or hypo-CpGs there is a clear enrichment of bystander modifications at sites with increasing positive (**k**,**m**), and negative (**l**,**n**) correlation with age (all *P* < 10^−15^, Kruskal–Wallis test).

As our age-associated CpGs were identified in blood, we have next aimed for epigenetic modulation in primary T cells. We again co-transfected guide RNAs that target age-hypermethylated CpGs (*PDE4C, FHL2, ELOVL2, KLF14* and *TEAD1*; *n* = 3; controls with scramble guide RNA and dCas9-DNMT3A; Fig. 5a). In fact, after 21 days we observed site-specific DNAm, particularly at the target sites of *FHL2* and *KLF14* (Fig. 5b). Notably, hypo- as well as hypermethylated bystander modifications were enriched at CpGs that gain or lose DNAm with aging in blood, and this was particularly observed at age-hypermethylated CpGs (Fig. 5c,d). Thus, the induction of DNAm at CpGs that gain DNAm with age did not have complementary effects on age-hypo and age-hypermethylated CpGs, but bystander effects were clearly enriched at these sites.

**Extended Data Fig. 7:**
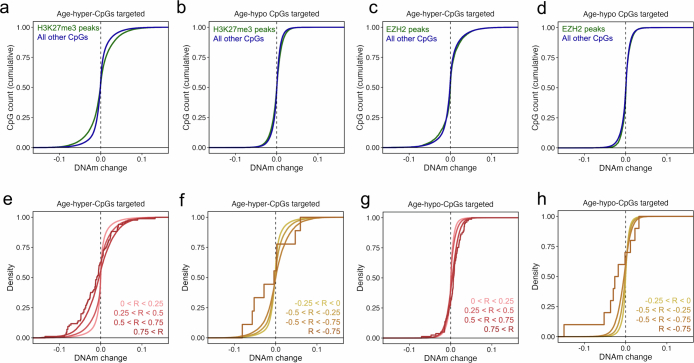
Association of bystander effects in T cells. **a,b**) Cumulative distribution functions comparing DNAm profiles at H3K27me3 peaks (GSE176621: H3K27me3 Mint-ChIP-seq of T cell male adult; 69806 CpGs) and all other CpGs (*P* values < 10^−15^, two-sided, two-sample Kolmogorov–Smirnov-test). **c,d**) Cumulative distribution functions comparing DNAm profiles at the top 4764 CpGs associated to EZH2 binding sites (GSE253773: EZH2 CHIP-seq of CD4 T cells). There are hardly any changes in DNAm patterns at EZH2 target sites. **e-h**) Cumulative distribution functions corresponding to Fig. 5k–n. The bystander modifications upon epigenetic editing at either age-hypermethylated CpGs or age-hypomethylated CpGs were clearly enriched at CpGs with increasing positive correlation (e,g), or decreasing negative correlation (f,h) with chronological age (R).

Analogously, we next targeted DNAm at the five age-hypomethylated CpGs at *COL1A1, AKAP8L, CSNK1D, MEIS1-AS3* and *IGSF11* (*n* = 3; Fig. 5e). After 10 days, we did not observe clear DNAm changes in the genome-wide DNAm profiles at the target regions, and there were only very moderate bystander modifications (Fig. 5f). These results further support the notion that epigenetic engineering was more stable at the age-hyper-CpGs than at the age-hypo-CpGs. Accordingly, the genome-wide aging signature was less affected with transient manipulations of age-hypomethylated CpGs, but there were still moderate enrichments (Fig. 5g,h). It needs to be noted that culture expansion of T cells also entails DNAm changes^30^, but we did not observe a coherent effect on these signatures and always compared controls with the same culture time. Under the assumption that Polycomb targets are more prone to change upon epigenetic editing, we used public chromatin immunoprecipitation sequencing (ChIP-seq) datasets and found significant enrichment of H3K27me3 high regions at bystanders^31^, whereas no significant enrichment was observed for EZH2 targets^32^ (Extended Data Fig. 7a–d).

As both epigenetic engineering-approaches at either age-hyper or age-hypomethylated CpGs were clearly enriched at age-associated genomic regions, we have subsequently analyzed how this might impact epigenetic clocks. To this end, we have utilized eight different epigenetic algorithms that have been trained for blood samples: Zhang^33^, Vidal-Bralo^34^, Lin^4^, Horvath 1 (multi-tissue)^10^, Horvath 2 (skin and blood)^35^, Hannum^29^, PhenoAge^7^ and updated PhenoAge^7^. In fact, all these epigenetic clocks revealed an epigenetic age-acceleration of up to 10 years after epigenetic editing at the age-hypermethylated regions in comparison to the non-targeted dCas9-DNMT3A (Fig. 5i); however, it must be noted that the previously established epigenetic clocks also comprise some of our target CpGs, which may contribute to the consistent age-acceleration. In contrast, the transient effects at age-hypomethylated regions had a less coherent impact on epigenetic clocks (Fig. 5j). Furthermore, the bystander modifications upon either targeting age-hyper or age-hypomethylated CpGs were simultaneously observed at age-hyper and age-hypomethylated CpGs, indicating that there is rather no directed effect on epigenetic age predictions.

To better understand if the epigenetic editing approaches preferentially target CpGs with higher age-association in T cells, we have utilized datasets of purified T cells of four studies^36–39^ to determine the correlation with chronological age for each CpG. In fact, with increasing correlation there was further enrichment of hypo- and hypermethylated bystander effects, and this was observed for age-hyper and age-hypomethylated CpGs at the same time (Fig. 5k–n and Extended Data Fig. 7e–h).

### Epigenetic editing in mesenchymal stromal cells

**Fig. 6:**
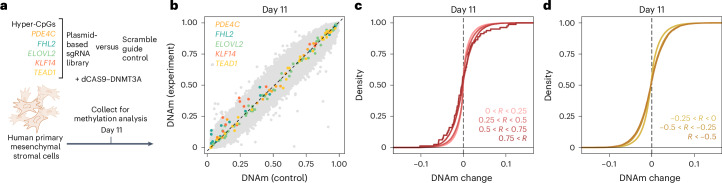
Epigenetic editing at age-hypermethylated CpGs in mesenchymal stromal cells. **a**, Scheme of multiplexed epigenetic editing in MSCs at five genomic regions that gain DNAm with aging. **b**, Scatter-plot of DNAm changes across the three biological replicas. CpGs associated to targeted genes (10-kbp region) are highlighted in the corresponding color. While there is a maximum gain of DNAm at a CpG in *PDE4C*, the modifications are lower overall and not equal across all CpGs in the target region. **c**,**d**, Cumulative distribution functions of DNAm profiles at CpGs that gain or lose DNAm with aging in T cells. Bystanders increase for strata with higher age-relatedness (positive correlation: *P* < 10^−8^; NS, negative correlation; Kruskal–Wallis test).

**Extended Data Fig. 8:**
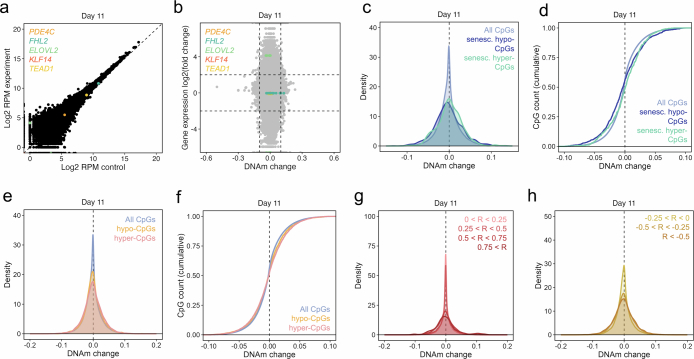
Enrichment of bystander modifications in mesenchymal stromal cells. **a,b**) Transcriptomic changes upon epigenetic editing in MSCs was analyzed for two of the three donors in independent experiments. a) The scatter-plot depicts reads-per million (RPM) in control *versus* experiment. b) Comparison of DNAm changes (n = 3) and transcriptomic changes (n = 2) revealed no clear association between DNAm changes and gene expression changes. **c,d**) In our previous work, we have identified 646 CpGs that gain and 2442 CpGs that lose DNAm during culture expansion of MSCs^41^. Since these CpGs may be associated with replicative senescence, they are referred to as senescence-hyper and senescence-hypo, respectively. We have analyzed if these CpGs are enriched in bystander modifications upon epigenetic editing at age-hypermethylated sites in MSCs. a) Gaussian kernel density estimate and b) cumulative distribution function demonstrates that bystander effects are slightly enriched in senescence-hyper (P < 10^−06^) and senescence-hypo-CpGs (P < 10^−9^, two-sided, two-sample Kolmogorov–Smirnov-test). **e,f**) Gaussian kernel density estimate and cumulative distribution function comparing the entirety of CpGs from the BeadChip with age-associated CpGs in blood. Bystander effects are significantly enriched at age-hypermethylated sites (P < 10^−15^, two-sided, two-sample Kolmogorov–Smirnov-test). **g,h**) Density plots of gaussian kernel density estimate of bystanders at CpGs that gain or lose DNAm with aging in T cells, corresponding to Fig. 6c, d.

To further validate the preferential bystander modifications at age-associated CpGs in another primary cell type, we subsequently performed epigenetic editing of mesenchymal stromal cells (MSCs). The same five genomic regions in *PDE4C, FHL2, ELOVL2, KLF14* and *TEAD1* were targeted with dCAS9-DNMT3A in MSC preparations of three different donors (Fig. 6a; *n* = 3; controls with scramble guide RNA and dCas9-DNMT3A). EPICv2 BeadChip analysis after 11 days post-transfection demonstrated up to 18% increase of DNAm at CpGs in *PDE4C*, *FHL2* and *KLF14* (Fig. 6b). There was no clear association between bystander modifications and transcriptomic changes (Extended Data Fig. 8a,b). Furthermore, long-term culture of MSCs entails senescence-associated with DNAm changes, which reveal moderate overlap with age-associated DNAm changes^40,41^. In fact, the bystander modifications in MSCs increased at CpGs that gain or lose DNAm with long-term culture (Extended Data Fig. 8c,d).

To test whether the bystander modifications were again enriched at other age-associated CpGs we again utilized the above-mentioned selection of age-associated CpGs based on blood samples^25^. The 10,000 hyper- and hypomethylated CpGs were both enriched in bystander modifications (Extended Data Fig. 8e,f). Furthermore, the age-associated CpGs in T cells again validated that those CpGs with higher correlation with chronological age were increasingly enriched in bystander modifications and this was observed particularly at CpGs that gain DNAm with age (Fig. 6c,d and Extended Data Fig. 8g,h).

## Discussion

**Extended Data Fig. 9:**
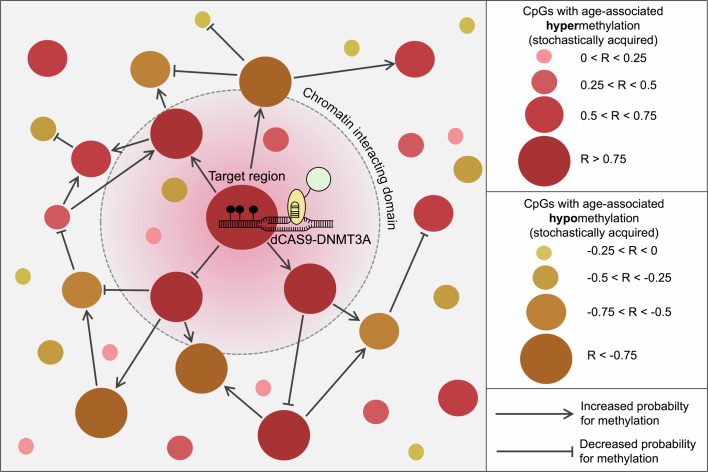
Hypothetical model of the epigenetic aging network. The findings of this study indicate that epigenetic editing at age-hypermethylated CpGs, utilizing dCAS9-DNMT3A or CRISPRoff, leads to epigenetic bystander modifications that are notably enriched at other age-associated CpG sites—specifically, those CpGs that either gain or lose DNA methylation with aging. A similar phenomenon was observed when targeting hypomethylated CpGs. These bystander modifications appear to be concentrated in open chromatin regions within interacting chromatin domains. This suggests the existence of an epigenetic network in which differentially methylated regions can communicate with one another. Although the exact mechanism underlying this interaction remains unclear, such connections may contribute to the stabilization of the epigenetic state and facilitate coherent epigenetic modifications. At the single-cell level, we propose that age-associated DNAm is influenced not only by stochastic changes at individual CpGs but also by a genome-wide crosstalk of epigenetic modifications.

It is largely unclear how DNA methyltransferases are guided to specific sites in the genome. The enormous complexity of these genome-wide epigenetic modifications suggests that they might be controlled by some kind of interactive network, possibly involving other epigenetic features, such as the histone code and chromatin architecture. The results of this study demonstrate that targeted epigenetic editing not only unravels the relevance of site-specific DNAm patterns at the target site, but also opens new opportunities to elucidate mechanisms that govern genome-wide epigenetic interactions (Extended Data Fig. 9).

While it has previously been demonstrated that epigenetic modifications can be stable for several months, and even during myeloid differentiation in a xenogeneic transplantation model^42,43^, other studies have reported transient effects of DNMT3A-mediated epigenome editing^14,15^. Our results indicated that epigenetic editing is particularly stable at genomic regions that gain methylation with age. It is conceivable that hypermethylated epigenetic clock sites are more susceptible to accumulate DNAm, whereas hypomethylated clock sites are more prone to lose DNAm. This may also be the reason why they consistently alter DNAm with age.

Furthermore, we assumed that successful targeting of the methyltransferases to a specific DNA strand would result in homogeneous methylation at that region, but bisulfite amplicon sequencing demonstrated otherwise. We have previously shown that also during aging, age-associated DNAm is not homogeneously acquired at neighboring CpGs^27^. In fact, recent reports demonstrate that large parts of the predictive accuracy of epigenetic clocks can be explained by stochastic processes^44,45^; however, it remains unclear why this stochastic drift occurs preferentially at specific age-associated CpGs. It is conceivable that DNAm patterns are changing in a much more dynamic manner toward a region-specific equilibrium than generally anticipated. This might explain why we even observed a gain in DNAm over time at some CpGs within the *PDE4C* amplicon, resulting in a more homogeneous pattern at neighboring CpGs.

Off-target effects have been described as a major problem for epigenome editing^46^. The footprints of CRISPR-guided DNAm approaches have been analyzed in detail before^18^ and it has been suggested that this is particularly based on the utilized targeting tool^23^. Notably, some of these off-targets were even hypomethylated, particularly at repetitive sequences and the majority of the off-targets were not associated with sequence homologies of gRNAs^18,23^. There have been attempts to decrease off-target effects, for example by different methyltransferases or targeting constructs^47^; however, the findings of our current study indicate that the off-target effects might rather reflect controlled epigenetic changes with a network: (1) These DNAm are observed in a highly reproducible manner in independent biological replicas; (2) they are observed in a very similar manner with different CRISPR constructs; (3) they include significant gains and losses of DNAm; (4) footprinting was also observed when we normalized the experiments for controls with epigenome editors and scrambled guide RNA; (5) bystander effects were not related to sequence homology of the guide RNAs; and (6) bystander effects were increasing with the age-relatedness of the CpGs. Therefore, we suggest that these modifications should rather be termed bystander modifications than off-target effects.

So far, it is unclear how bystander modifications might be regulated. It has recently been demonstrated that epigenetic editing at genes commonly methylated in cancer, including *CDKN2A*, results in repression of the corresponding p16 transcript and thus prevents cells from engaging senescence arrest^42^. In contrast, we observed a nonsignificant upregulation of *PDE4C* upon targeting *PDE4C*, which might be due to the fact that the age-associated region is not in the promotor^48^. Either way, it is unlikely that differential gene expression of target genes mediates epigenetic networks, because *PDE4C* expression did not reveal clear age-association in previous studies (for example *R* = 0.32 in ref. ^49^) and there is no rationale as to how their gene expression directs DNAm to specific sites in the genome. We therefore speculate that there is a more direct mode of interaction that can assimilate or dissimilate DNA methylation patterns across the genome. Our 4C-sequencing results indicate there might be chromatin interaction between genomic sites that have coherent bystander modifications; however, this does not fully explain all the complex DNAm changes, and it is also unclear how the interaction in chromatin conformation is governed. Architectural proteins, such as CCCTC-binding factor (CTCF), can medicate intra- and interchromosomal interactions. We previously demonstrated that age-associated hypermethylation indeed peaks near CTCF binding sites^27^. Knockdown of CTCF did not interfere with epigenetic clocks^12,50^, but it is still conceivable that the formation of chromatin loops contributes to bystander modifications. Furthermore, histone modifications may be involved in the regulatory mechanism. Polycomb repressive complex 2 (PRC2) targets are enriched among the CpG sites that gain methylation with age and can even be used as a biomarker for epigenetic aging^32^. A comprehensive analysis of such additional modifications can provide valuable insight into the underlying mechanisms of the epigenetic network proposed in this study.

Perhaps the most notable finding of this study was that the bystander modifications of age-associated regions were significantly enriched at other age-associated CpGs throughout the genome. Hypermethylation with aging occurs preferentially at regions with high CpG density^51^, which may even be sufficient to predict aging trajectories^52^. In fact, bystander modifications were also found to be significantly enriched adjacent to CpG islands; however, this enrichment does not account for the observed effects at age-associated regions, as the phenomenon persisted even after normalizing for both CpG density and DNA methylation levels. More notably, the bystander modifications exhibited gains and losses of DNA methylation at age-associated CpGs, with a notable increase in this effect observed at CpGs with stronger age associations. Previous work identified that CpGs in a low CpG context with flank nucleotides A and T are prone to lose methylation with age^26^, which was also observed in our analysis. In contrast, our bystander modifications had rather opposite flank nucleotides. Thus, bystander modifications can not only be attributed to the above-mentioned site-specific characteristics, further pointing toward an epigenetic network.

Our results indicate that it is possible to interfere with age-associated DNAm patterns by epigenetic editing. While epigenetic editing at age-hypermethylated CpGs accelerated epigenetic age in T cells across various clocks, it is noteworthy that these clocks also include target regions and that at least some of the effect will be attributed to this. As both age-hyper and age-hypomethylated CpGs are simultaneously modulated by epigenetic editing, the impact on the epigenetic clocks largely depends on the specific signatures employed. Currently, it seems that directing epigenetic aging through editing is not feasible; nevertheless, a deeper understanding of the underlying network may eventually facilitate this. Furthermore, it is quite possible that other epigenetic processes occurring during development and disease are influenced by similar interactive epigenetic dynamics.

Taken together, our results indicate that epigenetic editing at age-associated regions can perturb an epigenetic network and evoke coordinated epigenetic bystander modifications. These modifications are enriched at other age-associated CpGs and therefore have impact on epigenetic clocks. While many questions remain unanswered, our findings open the perspective to eventually control epigenetic aging in the future and thereby answer the question of whether the epigenetic clocks are per se functionally relevant.

## Methods

### Collection of biomaterials

Primary human T cells were obtained from peripheral blood of blood donors at the Clinic of Transfusion Medicine, RWTH University Hospital Aachen. Samples were taken after written and informed consent following the procedure approved by the ethics committee of RWTH Aachen University (EK 206/09) and supported by the RWTH central biomaterial bank (cBMB). The donor age (years) and sex (female (f) or male (m)) was reported for experiments targeting age-hypermethylated sites (28-f, 35-m and 38-m) and age-hypomethylated sites (54-m, 60-m and 68-m). Primary human MSCs were isolated from bone-marrow samples of patients undergoing orthopedic surgery after written and informed consent (ethics approval EK300/13). There was no compensation for participating in this study.

### Constructs for epigenetic editing

We use two constructs for epigenome editing: (1) dCAS9-DNMT3A/3L coexpressed with enhanced green fluorescent protein (eGFP) for selection^13^ (Addgene, 128424); and (2) CRISPRoff that comprises the DNMT3A/3L domain at the N terminus, and blue fluorescent protein (tagBFP) with a KRAB domain at the C terminus (Addgene, 167981; Fig. 1a)^15^. Guide RNAs were ordered as dsDNA from Integrated DNA Technologies (Supplementary Table 1) and cloned into the expression vector (Addgene 44248) by double digest with BstXI and NotI (Thermo Fisher Scientific). Plasmids were amplified in DH5-α competent cells (EC0112, Thermo Fisher), and were isolated using an M + N Nucleospin Plasmid kit. Successful cloning was confirmed by enzymatic restriction and Sanger sequencing (Eurofins).

### Cell culture

HEK293T cells were cultured in Dulbecco’s modified Eagle medium (Gibco) with 10% FCS (Bio&Sell) and penicillin–streptomycin (Life Technologies) at 37 °C and 5% CO_2_ and passaged manually at 80–90% confluency. The identity of this cell line was reconfirmed by their DNAm profiles (Illumina BeadChip).

Peripheral blood mononuclear cells were collected by density gradient centrifugation, and T cells were isolated by magnetic separation with the human Pan T Cell isolation kit and stimulated using TransAct (both Miltenyi Biotec). Cells were then cultured in TexMACS medium (Miltenyi Biotec) with 10% FCS (Bio&Sell), IL-2 (50 IU ml^−1^, Gibco) and penicillin–streptomycin 100 U ml^−1^ (Gibco) at 37 °C and 5% CO_2_.

MSCs of three donors (32–63 years old) were cultured in DMEM low-glucose (Sigma-Aldrich) supplemented with pooled human platelet lysate (10%), l-glutamine (2 mM, Gibco), penicillin–streptomycin (100 U ml^−1^, Gibco) and heparin (5 IU ml^−1^, B. Braun) at 37 °C and 5% CO_2_^53^.

### Transfection and selection of cells

HEK293T cells were transfected with the TransIT-LT1 transfection reagent (Mirus Bio) in six-well plates with 1.25 μg of plasmids for the CRISPR-construct and 1.25 μg of plasmids for single gRNA. We either pooled two gRNAs targeting *PDE4C* or up to nine gRNAs for multiplexed editing. After 24 h we selected the transfected cells either by puromycin treatment (2 μg ml^−1^ for 2 days) or flow-cytometric sorting for double-positive cells (BD FACSAria fusion).

Transfection of T cells was performed using the NEON transfection system (Invitrogen). Then, 5 million T cells were transfected with 5 µg of plasmids for CRISPR constructs and 5 μg of gRNA plasmids inside of a NEON 100-μl pipette tip with one pulse (20 ms) at 2,100 V. After 24 h, cells were resuspended in MACS buffer for flow-cytometric sorting for double-positive cells (BD FACSAria fusion) and further cultured in TexMACS medium as indicated above for up to 21 days.

For transfection of MSCs 1 million cells, 2.5 μg of plasmids for CRISPR constructs, and 2.5 μg of gRNA plasmids were combined inside of a NEON 100-μl pipette tip and treated with one pulse (20 ms) at 1,700 V. Cells were cultured in the above-mentioned MSC culture medium, but without antibiotics for the first 24 h. After 48 h, cells were resuspended in MACS buffer for flow-cytometric sorting for double-positive cells (BD FACSAria fusion) and further cultured for 11 days.

### Analysis of DNA methylation profiles

Genomic DNA was isolated from HEK293T cells, primary T cells and primary MSCs using the Macherey-Nagel Tissue kit. DNA was bisulfite converted and hybridized to EPIC Illumina BeadChips (either v.1 or 2) at Life and Brain. Initial quality control was performed on IDAT files using the minfi package (v.1.48.0)^54^ and for subsequent preprocessing we used the sesame package (v.1.20.0). Data were normalized with noob and probes with a detection *P* > 0.05 in at least one sample were removed^55,56^. Furthermore, we excluded probes associated to X or Y chromosomes, SNPs and CpGs flagged in the b5-manifest (Illumina). Differential methylation analysis was performed using limma. Probes with a *P* value < 0.05 (Benjamini–Hochberg adjusted) and at least 10% mean difference in DNAm were considered significantly differentially methylated. Genomic feature annotation and flank sequences (hg38 genome) were obtained from the manifest file EPICv2 (Illumina), which uses the chromosomal coordinates of islands from UCSC. Shores were 0–200 bp distant and shelfs were 2–4 kbp distant from islands. Enrichment of CpGs in genomic features was analyzed using chi-squared tests. Significance between mean DNAm changes of subgroups was estimated by unpaired *t*-tests. All plots were generated in R using ggplot2 and ggforce.

Age-associated CpGs in blood were based on a large-scale epigenome-wide association study^25^. Alternatively, we identified T cell age-associated CpGs in four datasets of purified cells (healthy donors of CD4^+^ or CD8^+^ T cells from datasets GSE59065 (ref. ^36^), GSE56581 (ref. ^37^), GSE110554 (ref. ^38^) and GSE34639 (ref. ^39^)). The already preprocessed methylation data was downloaded by using the getGEO function from the geoquery package in R. Probes associated with X or Y chromosomes and single-nucleotide polymorphisms (SNPs) (according to the Illumina annotation files) were excluded. Only probes present in all three 450k, EPICv1 and EPICv2 methylation arrays were considered. The modified targeted sites as well as their neighboring sites (in a 10-kbp window) were excluded from the analysis.

### Pyrosequencing

Genomic DNA was bisulfite converted with the EZ-DNA methylation kit (Zymo) and PCR amplified at the age-associated regions with the PyroMark PCR kit (QIAGEN). Then, 10 μl of PCR-product and 2 µl of sequencing primer (4 μM) were sequenced on a Pyromark Q48 Autoprep (QIAGEN), as described previously^27^. Primer sequences are provided in Supplementary Table 2.

### Bisulfite barcoded amplicon sequencing

Bisulfite barcoded amplicon sequencing at age-associated genomic regions was performed as described in detail previously^27^. In brief, bisulfite-converted genomic DNA was amplified with the PyroMark PCR kit (35 cycles; QIAGEN) with primers containing handle sequences. The amplicons were purified with the Select-A-Size DNA Clean & Concentrator Magbead kit (Zymo) and further amplified with a second PCR (ten cycles) to add barcodes, i5 and i7 adaptors. The PCR products were pooled equimolarly and washed in a size-selection column (Zymo Select-A-Size DNA Clean & Concentrator). Sequencing was performed using the Illumina v2 Nano reagents and flow cell kit with 50% PhiX control on MiSeq (250 bp, paired end). PCR primers are provided in Supplementary Table 3. We used Bismark to obtain methylation calls^57^ and excluded reads with at least one undefined methylation status. Results were visualized using ggplot in R or matplotlib and seaborn packages in Python.

### Chromatin conformation capture 4C analysis

Preparation of the i4C templates was performed as previously described^58^. In brief, 10^7^ HEK293T cells were used for isolation of nuclei in a near-physiological isotonic buffer. The primary restriction enzyme digestion was performed with 800 U *Nla*III or *Apo*I (New England Biolabs, R0125 and R3566). Following in situ ligation of digested chromatin, nuclei were lysed, and the isolated DNA was digested with *Cvi*QI (New England Biolabs, R0639), circularized by ligation and purified. The library was amplified by inverse PCR with viewpoints-specific primers (Supplementary Table 4) coupled to Illumina adaptors for sequencing. Following inverse PCR, the i5 and i7 Illumina adaptors were used to generate NGS-compatible libraries as previously described^59^. Amplicons were sequenced in a 50-bp paired-end mode on the Illumina NextSeq2000 platform (Genomics Facility, Erasmus MC Rotterdam). Viewpoint primers sequences were trimmed using cutadapt, and the remaining sequences were aligned to the reference genome (hg38) by HISAT2. After quantification of reads per fragment, 4C data were aligned with the positions of the Illumina BeadChip (Illumina manifest file, hg38) using the GenomicRanges package in R. Co-localization of CpGs within 250 bp of tolerance around the 4C-seq reads was considered an overlap. The number of overlaps for age-hyper- and age-hypomethylated sites and the entirety of CpGs on the EPIC-array were calculated. Odds were calculated as overlapping CpGs by non-overlapping CpGs. Odds were normalized by odds of the entirety of CpGs on the microarray. We further analyzed the association using chi-squared testing and logistic regression.

### Gene expression analysis

RNA was isolated using the RNA plus kit (Macherey-Nagel). Library preparation (QuantSeq 3′ mRNA library prep, Lexogen) and sequencing (Novaseq 6000, 100 bp single end) was performed at Life and Brain. Preprocessing of fastq files was carried out using the nextflow-core pipeline. Adaptors and low-quality reads were removed using Trim Galore. We used STAR for alignment (hg38 genome) and count matrices were generated by Salmon. Counts matrices were normalized and differential gene expression was performed using DESeq2 in R^60^. *P* values were calculated using a negative binomial GLM function and Wald test. Differential gene expression was considered significant with a *P* value <0.05 (Benjamini–Hochberg adjusted) and at least a twofold change. For integration of transcriptomic changes and DNAm changes, BeadChip probes were matched to transcripts based on Gene ID.

### Statistics and reproducibility

The number of biological replicas per experiment are indicated by *n*. There were no statistical methods used to determine sample size. Sample sizes were similar to those reported in previous studies with comparable experiments^14,15,42^. All attempts at replication were successful. The study was not subjected to randomization because cell lines or primary cells were split equally to control and experimental conditions before every transfection experiment. Data collection and analysis were not performed blinded to the conditions of the experiments. One replica of T cells failed quality control for Illumina EPIC data (experiment and control cells), due to the low amount of DNA. Thus, this sample was not used for further analysis and the experiment was repeated to obtain the third replicate. All experiments were conducted in the same laboratory, and control and experimental conditions were subjected to identical treatment except for the plasmid DNA input (intended intervention). *P* values were calculated using the stats package in R (v.4.3.2) if not described otherwise. Extremely small *P* values are reported as *P* < 10^−15^. The statistical tests are noted in the figure legend and described in the Methods (*t*-tests, chi-squared tests, Pearson correlation, Kolmogorov–Smirnov-test and Kruskal–Wallis test). Normal distribution of data was not formally tested, but distribution functions of the respective data are provided.

### Reporting summary

Further information on research design is available in the Nature Portfolio Reporting Summary linked to this article.

## Supplementary information

Supplementary Information Supplementary Tables 1–4.

Reporting Summary

## Data Availability

DNA methylation profiles, mRNA-seq and i4C-seq data generated in this study can be accessed from the Gene Expression Omnibus under the series number GSE269760. We also used the following publicly available datasets: hg38 reference genome by UCSC (Illumina EPIC manifest files), ATAC-seq data (GSE108513)^28^, ChIP-seq data (GSE176621 (ref. ^31^) and GSE253773 (ref. ^32^), and EPIC DNAm profiles of T cells (GSE59065 (ref. ^36^), GSE56581 (ref. ^37^), GSE110554 (ref. ^38^) and GSE34639 (ref. ^39^)). All other data supporting the findings of this study are available from the corresponding author upon request.
