# Supplementary material for: Epigenetic editing at individual age-associated CpGs affects the genome-wide epigenetic aging landscape
Source: Nat Aging. 2025 Mar 24;5(6):997–1009. doi: 10.1038/s43587-025-00841-1 (PMC12176646; doi:10.1038/s43587-025-00841-1)
Supplement: Supplementary file 1 — Supplementary Tables 1–4. [file 43587_2025_841_MOESM1_ESM.pdf]

nature aging

Supplementary information https://doi.org/10.1038/s43587-025-00841-1

Epigenetic editing at individual age-associated CpGs affects the genome-wide epigenetic aging landscape

In the format provided by the authors and unedited

Table of Contents

Supplemental table S1: Guide RNA sequences for age-associated hyper-CpGs 2
Supplemental table S2: Primers for pyrosequencing 3
Supplemental table S3: Primers for bisulfite amplicon sequencing 3
Supplemental table S4: Primers for i4C sequencing.. 4

Supplemental table S1: Guide RNA sequences for age-associated hyper-CpGs

Target gene and no.	sgRNA-guide sequence	Target CpG ID (Illumina BeadChip)
Hypermethylated with age:		
PDE4C #1	5‘-TCTGGCGCATGGAGAACCTG-3‘	cg17861230
PDE4C #2	5‘-TCGGATCCGGACAAGTCCGC-3‘	cg17861230
ELOVL2 #1	5‘-GACGAAACGGCCCCGAGGCT-3‘	cg16867657, cg21572722
ELOVL2 #2	5‘-GGGGAGAAGCAGTATCGTGC-3‘	cg16867657, cg21572722
FHL2 #1	5‘-GAGAAGCCCCAGGACGTGCCG-3‘	cg22454769, cg06639320
FHL2 #2	5‘-GACTGTGCTCCCAAGACCCGG-3‘	cg22454769, cg06639320
KLF14 #1	5‘-GCAACCCAGAAGTTCCGACTG-3‘	cg22285878
KLF14 #2	5‘-GACGGAGCACGGGATCGGGT-3‘	cg22285878
TEAD1 #1	5‘-GCACAAGTCTGCGCAGACCC-3‘	cg04940570
Hypomethylated with age:		
MEIS1-AS3 #1	5‘-CAAGTGAATAACTACTCAGC-3‘	cg11807280
COL1A1 #1	5‘-TAAGATTGGAGAAGGTTGAC-3‘	cg18618815
COL1A1 #2	5‘-TCTCCTATAGAAGAACTCCC-3‘	cg18618815
AKAP8L #1	5‘-CACACGTGTTAAGTACTCTT-3‘	cg25533247
CSNK1D #1	5‘-GCCTTACCTCCTGACACCTA-3‘	cg19761273
IGSF11 #1	5‘-TAATCAATAAGACCTTCCTA -3‘	cg00329615
IGSF11 #2	5‘-GGCTACGATTCAGGGACACC-3‘	cg00329615

Supplemental table S2: Primers for pyrosequencing

Gene name	Label	Primer sequence
PDE4C	Forward	5'-AGGTTTGTAGTAGGTTGAG-3';
	Reverse	Biotin-5'-AACTCAAATCCCTCTC-3'
	Seq-Primer	5'-GTTATAGTATGATTAGAGTTT-3'
FHL2	Forward	5'-GTGTTTTTAGGGTTTTGGGAGTATAGTAGT-3'
	Reverse	Biotin-5'-CACCTCCTAAAACTTCTCCAATCTCC-3'
	Seq-Primer	5'-GGTTTTGGGAGTATAGTAGTT-3'
ELOVL2	Forward	5'-Biotin-GGGAGGGGAGTAGGGTAAGTGA-3'
	Reverse	5'-CCATCTAAACAACCAATAAATATTCCTAAA-3'
	Seq-Primer	5'-AATAAATATTCCTAAAACTC-3'
MEIS1-AS3	Forward	5'-ATTTTTGTTTGAAGGTTTTTATAAAATATG-3'
	Reverse	5'-ACCTTTAAAACAACAAAATAAATCACACT-3'
	Seq-Primer	5'-ACCATACTTAACATCCA-3'
AKAP8L	Forward	5'-TGAAGTTTGGAATTTATGATTTGTTTAAG-3'
	Reverse	Biotin-5'-CCCAAAAAACAAACTAAAAACATAACTAAT-3'
	Seq-Primer	5'-GAGAGATTTTGTAATAGTGTA-3'
CSNK1D	Forward	5'-GGAGGTTTTGATGTTTAGTTTGAAGAT-3'
	Reverse	Biotin-5'-CAAATCCAACACAAATAAAAATATTAACTC-3'
	Seq-Primer	5'-GGTTAGATTATTTGTTTTTTTTTAG-3'
COL1A1	Forward	5'-TTGAAGGGAAGAGGTAAGGAAGATTTTA-3'
	Reverse	Biotin-5'-TAACCCATCTTTTTCCTTCTTCTCA-3'
	Seq-Primer	5'-AATTTGTATAGAGAGTGTTTATTG-3'
IGSF11	Forward	5'-GTTGGATAGTTTGTGGGTAGAAAATTTA-3'
	Reverse	Biotin-5'-ATTATTCATTCATTATTCTCCTTAAAAAAATCTTATT-3'
	Seq-Primer	5'-AGAAGTTAAGAAGGTATAGATA-3'

Supplemental table S3: Primers for bisulfite amplicon sequencing

Gene Name	Label	Primer sequence
PDE4C, 1st PCR	Forward	5'-CTCTTTCCCTACACGACGCTCTTCCGATCTTATGGAGAATTTGGGG-3'
	Reverse	5'-CTGGAGTTCAGACGTGTGCTCTTCCGATCTCTACAAAAAACCCCTACC-3'
Adapters, 2nd PCR	i5	5'-AATGATACGGCGACCACCGAGATCTACACTCTTTCCCTACACGACGCTCTTCCGATCT-3'
	i7	5'-AGATCGGAAGAGCACACGTCTGAACTCCAGTCAC[Barcode]ATCTCGTATGCCGTCTTCTGCTTG-3'

Supplemental table S4: Primers for i4C sequencing

Primer	Location (hg38)	Adapter primer Illumina; view-point specific sequence
Pri-PDE4C-NlaIII	chr19:18233097-18233114	5‘-TCGTCGGCAGCGTCAGATGTGTATAAGAGACAG-3‘; 5‘-AGGTGCTTCGGGGCTCTG-3‘;
Sec-PDE4C_CviQI	chr19:18232652-18232672	5‘-GTCTCGTGGGCTCGGAGATGTGTATAAGAGACAG-3‘; 5‘-CCAGATGTGTTTGGGGTGCTC-3‘
Pri-FHL2-NlaIII	chr2:105399436-105399455	5‘-TCGTCGGCAGCGTCAGATGTGTATAAGAGACAG-3‘; 5‘-AGGGGGTCACTTCTCAGGAG-3‘
Sec-FHL2_CviQI	chr2:105399228-105399247	5‘-GTCTCGTGGGCTCGGAGATGTGTATAAGAGACAG-3‘; 5‘-AAGAAAGGAGCCCTGGCAAA-3‘
Pri-MEIS1-ApoI	chr2:66427399-66427383	5‘-TCGTCGGCAGCGTCAGATGTGTATAAGAGACAG-3‘; 5‘-GAAGGCTTCCTGCGGCG-3‘
Sec-MEIS1_CviQI	chr2:66427579-66427598	5‘-GTCTCGTGGGCTCGGAGATGTGTATAAGAGACAG-3‘; 5‘-AGTGACTAGAGCACGTTCGC-3‘
